# Mother and Child Emotion Regulation: A Moderated Mediation Model

**DOI:** 10.3390/children12020175

**Published:** 2025-01-30

**Authors:** Maria Cenușă, Maria Nicoleta Turliuc

**Affiliations:** Faculty of Psychology and Educational Sciences, Alexandru Ioan Cuza University of Iasi, 700554 Iasi, Romania; turliuc@uaic.ro

**Keywords:** child emotion regulation, maternal playful behavior, maternal emotion regulation, paternal empathy

## Abstract

The relationship between mother and child emotion regulation (ER) is widely researched, but fewer studies have investigated explanatory variables or those affecting the strength of this link. Background/Objectives: The present study focused on maternal play behavior, considered as an explanatory mechanism between mother and child ER. In addition, the study explored the moderating role of paternal empathy in the association between maternal emotion regulation (ER) and maternal play behavior. Methods: This present cross-sectional study involves 103 mothers and their husbands (the children’s fathers), with at least one child between the ages of 3 and 6 who is typically developed. Results: Our findings show that maternal play behavior mediates the relationship between maternal cognitive reappraisal (CR), expressive suppression (ES), and child ER. Regarding the moderating role of paternal empathy, lower levels moderate the association between ER and maternal play behavior. Conclusions: Our findings highlight the importance of maternal play behavior as an explanatory variable between maternal and child outcomes and, concomitantly, of paternal variables, such as empathy, in supporting maternal play behavior when it comes to achieving better child outcomes.

## 1. Introduction

Emotion regulation (ER) refers to the capacity to influence one’s emotions and experiences across a lifespan [1]. In children, ER involves the child’s capacity to handle their own emotions and communicate them appropriately to meet various demands in social settings, with this ability being associated with the optimal management of emotions in the context of frustration, psychological well-being, and successful relations with peers [2,3]. We focused on preschoolers because early childhood ER lays the foundation for future adulthood ER [4,5]. Guided by the Family Systems Theory (FST), which underlines the interdependence and mutual influences among family members [6,7], this research included children’s mothers and fathers. According to the Tripartite Model [8], during early childhood, parental ER, along with parental behavior, is a critical factor in shaping children’s ER [9]. A relevant difference across emotion regulation is whether the emotion regulation goal is activated in the individual who has (or could have) an emotional episode. Extrinsic emotion regulation involves regulation applied to others’ emotional episodes in which a person tries to regulate another person’s emotions (e.g., the mother’s attempts to calm an angry child). In contrast, intrinsic emotion regulation involves regulation applied to one’s emotional experience (e.g., when the child tries not to appear sad) [10,11]. The quality of parental ER is critical because preschool children remain greatly dependent on extrinsic ER provided by their parents, even as they increasingly use intrinsic ER [12,13].

The relationship between maternal ER and child ER is well documented in the literature, but fewer studies have investigated the intermediary variables explaining or affecting the strength of this link. Therefore, maternal playful behavior may explain the association between mother and child ER through the lens of the Attachment Theory [14]. Specifically, playful parenting may foster a secure attachment through greater parent–child closeness, which is further related to children’s functional ER strategies [15,16]. According to John and Gross [5], we focus on two common ER strategies: cognitive reappraisal and expressive suppression. Cognitive reappraisal (CR) involves changing a potentially emotional situation to alter its meaning and modify its emotional consequences, and it is associated with positive psychological outcomes (e.g., more experience and greater expression of positive emotions as well as more social closeness). In contrast, expressive suppression (ES) refers to the attempt to inhibit the ongoing emotion-expressive behavior correlated with adverse outcomes at the emotional, cognitive, and social levels (e.g., greater negative emotion experience, poorer memory for social information, and lack of emotional closeness with others) [5].

Addressing the gaps in the literature, we aimed to explore the mediating role of maternal playful behavior in the relationship between mother and child ER in a community sample including nonclinical parents and their preschool children. We also tested the moderating influence of fathers’ empathy in the relationship between maternal CR, ES, and maternal playful behavior.

### 1.1. Mother’s ER and Child’s ER

Children learn to use their parents’ ER strategies, namely emotional reappraisal or suppression [17,18], and in doing that, they do not simply internalize their parents’ ER styles but rather develop their capacity for ER [19]. Child ER is related to a greater extent to the mother’s ER than the father’s ER, considering that the mothers are the primary caregivers and socializers of emotions among their children, with the fathers’ parental role being less scripted than the maternal ones [20]. Specifically, in Romania, traditionally, mothers are more caring, while fathers are seen as the breadwinners [21].

The challenging and overwhelming character of childrearing stems from the fact that parents strive to develop their child’s emotional regulation while managing their own emotions [18,22]. Overall, the studies investigating the association between parent ER and children ER show that CR is positively associated with children’s ER, whereas ES is negatively associated with children’s ER [23,24]. Thus, we tested whether in our sample the mother’s ER is significantly associated with their child’s ER.

### 1.2. Maternal Playful Behavior as a Mediator

Although the literature does not clearly define adults’ playfulness, recent studies have defined playfulness as a personality trait (associated with play as the actual behavior) that allows individuals to interpret everyday situations in a way that makes them interesting, mind-provoking, and engaging [25,26].

One of the most positive parenting behaviors that is more appropriate during early childhood is represented by parental playful behavior [13,15]. Although it is known that playful parent–child interactions are connected with many children’s socio-emotional positive outcomes [27,28], the contribution of parents’ playful behavior to their children’s optimal functioning has received little attention [15]. Indeed, previous studies investigated, to a higher extent, children’s playfulness [29] but not parental playfulness understood as parental playful behavior, with few exceptions [30,31], as a predictor of children’s socio-emotional outcomes.

Previous studies showed that parents who use CR are more engaged in playful interaction with their children, since both CR and parental playful behaviors involve the parents’ ability to reframe different events and interactions with their child in a playful manner [15,32]. Regarding ES, it has been associated with a decreasing expression of positive emotions, less success in reframing negative situations, inauthentic interactions with others, or lower levels of emotional intimacy [33]. Therefore, suppressing parents engage in fewer playful interactions with their children, and they are less able to handle conflicts that arise during playful interactions, which manifest in less guidance and responsiveness and an overall lower quality of interaction [34]. As such, all of these characteristics of mothers using ES are opposite to those that parental playful behaviors encompass, such as positive effects, fun, spontaneity, humor, and enjoyment [35,36].

Past research explained the association between other parental variables and children’s outcomes through parental playfulness, such as parental behavior and child negativity [37], as well as parental emotional availability and child behavioral problems [38]. The association between the mother’s ER and children’s ER through maternal playful behaviors is understudied, although previous studies have revealed the importance of maternal playfulness when it comes to children’s ER [15]. Specifically, researchers have hypothesized that children who are raised by playful mothers adopt different social roles that involve representations of other people’s mental states, a fact that sets up a trigger for children’s emotion regulation development [39]. In addition, compared to mothers, fathers are usually less attuned to children’s emotions during playful interactions, while mothers are more aware of children’s shared emotions in the context of play (i.e., mothers engage children in conversation about emotions such as sadness and anger) [39,40]. Therefore, we aimed to investigate the explanatory role of maternal playful behavior in the association between mother and child ER.

### 1.3. Partner’s Empathy as a Moderator

Empathy is a multidimensional construct that involves an emotional and cognitive response arising from the recognition of another’s state that is somewhat similar to how the other person feels, thinks, or might be expected to feel or think in a situation [41]. According to the dynamic model of paternal influences on children [42,43], the father’s characteristics influence a child’s outcomes through paternal involvement. A significant predictor of the father’s involvement is represented by paternal empathy [44]. Paternal empathy contributes to an increased positive moods in mothers and to a higher support and sensitivity toward mother and child needs. [45]. In line with this aspect, a study investigating the factors that impact playful mother–child interaction found that mothers who received higher levels of emotional support from their partners, through empathy [46], reported more playful mother–child interactions [47]. The FTS includes mutual influence through their partners’ interdependence [48,49,50]. Therefore, according to the affective spillover effect, mothers who experience care and empathy in their relationships with their husbands may express similar qualities in relation with their children [51]. The extent to which mothers optimally manage their emotions to promote children’s ER through playful maternal behavior may depend on fathers’ empathy towards mothers confronted with difficulties and challenges in their parenting. Research that investigated fathers’ indirect influence on mothers is limited, with a few exceptions [52]. An investigation of the moderating role of paternal empathy in the association between maternal ER and maternal playful behavior is necessary considering fathers’ indirect influence on children through their relationship with their children’s mothers [53]. Empathy includes decoding others’ emotions and catching both positive and negative affects [54,55]. For instance, paternal cognitive empathy alleviates marital conflict and maternal depression produced by the mother’s parental stress [56]. As such, the interaction between paternal empathy and the mother’s ER creates the premises (e.g., close relationships and positive emotional experiences) for initiating and developing playful mother–child interactions. For mothers using CR, although they experience and express more positive emotions during playful interactions with their children, they are at risk of emotional overloading, considering that CR may come with important costs (e.g., reappraisal can reduce individuals’ motivation to solve problems) [57]. Regarding mothers using ES, they may feel more childcare pressure and a sense of being overwhelmed by their children’s emotional reactions, and they remain with lower levels of emotional regulation resources, knowing that suppressors exert additional efforts to manage emotional responses, which appear late in the emotion-generative process [58]. Therefore, we aimed to test whether paternal empathy has a moderating role in the association between maternal ER and play behavior.

#### The Present Study

This study examines the mediating role of maternal playful behavior in the association between mother and child ER, and the moderating role of paternal empathy in the relationship between mother ER and maternal playful behavior. Although previous studies have investigated the association between parents and children [59], the mediating role of maternal playful behavior in this association was insufficiently explored. The concept of playfulness was investigated mainly among children [60], most frequently kindergarten children [61], with few studies exploring the role of maternal playful behavior [15,39] in the relationship with other variables. Also, the role of paternal empathy when it comes to mother–child interaction needs further investigation.

Addressing the literature gap, we expanded this line of research in four relevant directions that are lacking in studies. First, the direct influence of maternal ER strategies on children ER in a non-clinical context is limited [62]. Second, the research on maternal playful behavior as an explanatory mechanism between mother and child ER is scarce, and many studies have investigated other explanatory mechanisms, such as parenting quality [63], parental reactions to children’s negative emotions [52,64], or family emotion expressiveness [65]. Third, previous research that included fathers explored the direct effects of paternal variables on children’s ER/socio-emotional development [66], although studies have shown that fathers can also indirectly influence children’s outcomes through the relationship that they have with their wives [53,67]. Fourth, many of the previous studies investigating parenting variables and child outcomes included fathers and mothers separately as parts of different dyads, with few exceptions [64]. As such, in line with theoretical models and the reviewed empirical results, we hypothesize the following:

**H1.**  *Mothers’ ER is significantly associated with children’s ER.*

**H2.**  *Maternal playful behavior mediates the association between maternal ER and child ER.*

**H3.**  *Paternal empathy moderates the relationship between mother ER and playful behavior.*

## 2. Materials and Methods

### 2.1. Participants

This current study comprises 103 mothers and their husbands (children’s fathers) from a sample of Romanian parents of preschoolers. The mothers completed a questionnaire between September and December 2022. In Table 1, the socio-demographic characteristics of the participants are presented. The parents met the following criteria: having at least one child between 3 and 6 years old who is typically developed without special needs.

### 2.2. Procedure

The protocol for this study was approved by the authors’ University Ethics Committee (approval code: 1013/17 May 2022), and the 2013 Helsinki Declaration ethical guidelines were followed when conducting the research. The participants were contacted through social media and online groups. Parents who had more than one child were asked to focus on the same child which met the following criteria: aged between 3 and 6 years, typically developed. Also, the parents were asked to read the explanation regarding the purpose of the study. After that, they were asked to sign an informed consent form. They were advised to complete the questionnaires, without consulting their partners, and to fill in a password (city/town and day/month/year of marriage), which was necessary to identify the parental dyad. Participation was voluntary, all information was anonymous and confidential, and the participants were free to withdraw at any time. The parents then completed all questionnaires online using the Google Forms software (https://www.google.com/forms/about/, accessed on 29 January 2025).

### 2.3. Measures

The mothers completed measures of ER, parental playful behavior, perception of children’s ER, and the socio-demographic questionnaire. The fathers completed only the measure of empathy and the socio-demographic items. The instruments were translated from English to Romanian by the authors.

Emotion regulation was assessed by the Emotion Regulation Questionnaire (ERQ) [33]. This self-reporting scale consists of 10 items which measure an individual’s tendency to use CR (e.g., “When I’m faced with a stressful situation, I make myself think about it in a way that helps me stay calm”) and ES (e.g., “When I am feeling positive emotions, I am careful not to express them”) to regulate emotions. The mothers rated statements on a scale ranging from 1 (strongly disagree) to 7 (strongly agree). The items within each subscale were averaged to provide scores for reappraisal and suppression, and higher scores indicate more reappraisal or suppression. In this study, the Cronbach’ alpha for the CR subscale was 0.87, and that for the ES subscale was 0.88.

Maternal playful behavior was assessed by the Parental Playfulness Questionnaire (PPQ) [15]. It is appropriate for parents of children aged 2–8. It includes 20 items, namely 13 direct items (e.g., “As a parent, I find that using games or humor helps me figure out and resolve parenting conflicts”) and 7 reversed items (e.g., “When I do things with my child, I am goal oriented and want to finish what I have planned without any interruptions”). The items are scored on a 5-point Likert scale (1—totally disagree; 5—totally agree). The final score is the sum of the 20 items, and higher scores reflect greater parental playfulness. The scale has high internal consistency, with the Cronbach’s alpha in our sample being 0.87 for the mothers.

The children’s emotion regulation was assessed using the Emotions Questionnaire for Parents (EQP) [68], which measures the parents’ perceptions of their children’s ability to regulate their emotions. The mothers assessed their children’s ability to regulate four emotions: sadness, anger, fear, and exuberance. The scale contains 20 items used to measure ER (e.g., “He/she has difficulties calming down on his/her own”) and 20 items used to measure emotionality. The mothers rated the statements on a scale from 1 (does not apply at all to my child) to 5 (applies very well to my child). In this study, only the ER subscale was used. The scores for the ER subscale were computed as the mean of the items’ scores, and higher total scores indicate better ER. The internal consistency of the ER scale in this study was 0.92 for mothers.

Empathy was measured using the Questionnaire of Cognitive and Affective Empathy (QCAE) [69]. The QCAE consists of 31 items comprising five subscales intended to assess cognitive (two subscales) and affective components of empathy (three subscales). The respondents used a 4-point Likert scale to indicate how much they agreed with each item’s statement (1—strongly disagree; 4—strongly agree). The final score is the sum of all the items; higher scores reflect greater empathy. The scale has high internal consistency, with the Cronbach’s alpha in our sample being 0.94 for the fathers.

The mothers and fathers reported their age, marital status, and educational level, as well as the child’s age and gender.

## 3. Results

### 3.1. Preliminary Analyses

In Table 2, the mean scores and standard deviations among study variables are presented. Skewness and Kurtosis were computed to verify the normality of the distributions. In Table 3, the correlations among the main study variables are presented, and CR is positively correlated with the child’s ER (*r* = 0.38, *p* < 0.01), with maternal playful behavior (*r* = 0.53, *p* < 0.01), and with the partner’s empathy (*r* = 0.40, *p* < 0.01), whereas ES is negatively correlated with the child’s ER (*r* = −0.33, *p* < 0.01) and with maternal playful behavior (*r* = −0.37, *p* < 0.01). The correlational analysis indicates that maternal CR is positively associated with the child’s ER, whereas maternal ES is negatively associated with the child’s ER. In addition, Pearson and Spearman correlations show that the following variables are significantly associated with some of the study’s main variables: the number of children and the child’s gender and age. Therefore, these variables were included as covariates in the subsequent statistical analyses.

### 3.2. Simple Mediation Analysis

**Figure 1:**
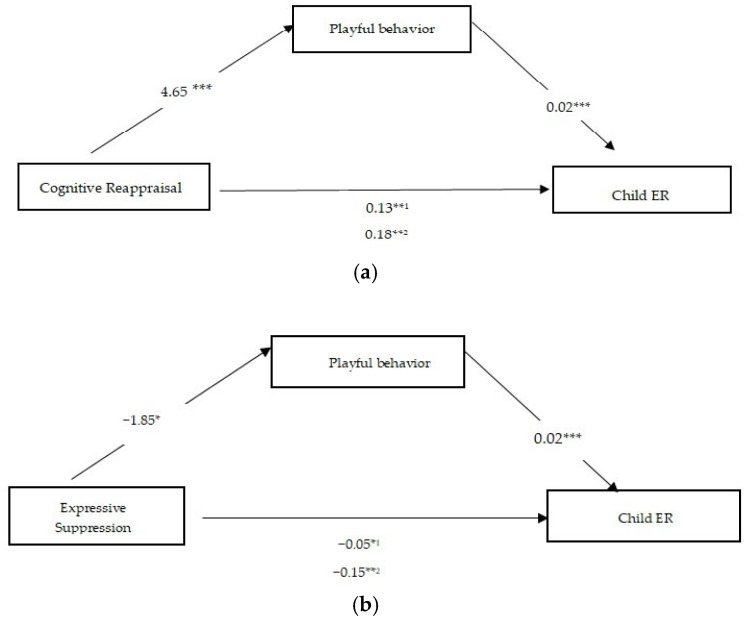
(**a**) Simple mediation model of link between mother’s CR and child’s ER, mediated by mother’s play behavior. ^1^ Indirect effect. ^2^ Total effect. *** *p* < 0.001. ** *p* < 0.01. (**b**) Simple mediation model of link between mother’s ES and child’s ER mediated by mother’s play behavior. ^1^ Indirect effect. ^2^ Total effect. *** *p* < 0.001, ** *p* < 0.01, and * *p* < 0.05.

To estimate the direct and indirect effects of mediation, we used Model 4 in the PROCESS macro v3.5 tool for IMB SPSS [70]. The results for maternal CR are presented in Figure 1a, and for maternal ES in Figure 1b.

A multiple regression analysis was conducted to estimate all components of the proposed mediation model. For maternal CR and ES, the results are displayed in Table 4. First, maternal CR was positively associated with the child’s ER (c = 0.18; *p* = 0.0012). Also, it was found that CR was positively related to maternal playful behavior (a = 4.65; t(4;98) = 6.74; *p* = 0.0000), and the mediator, maternal playful behavior, was positively associated with child ER (b = 0.02; *p* = 0.0001). The direct effect of the CR of a child’s ER failed to maintain a significant level (c’ = 0.04; *p* = 0.5016) after putting it into the model. The mediation variable of the mother’s play behavior indicates that the indirect effect of maternal CR on child ER is fully accounted for by the mother’s playful behavior. Lastly, the indirect effect was significant (a * b = 0.13; Boot SE = 0.0429; CI [0.0676; 0.2353]). Regarding, the mediating role of maternal playful behavior in the association between ES and children’s ER, the results show that ES has a significant negative effect on playful behavior (a = −1.85; SE = 0.7290; *p* = 0.0124), and after adding the mediation variable of mother’s playful behavior in the model, the effect of ES on child ER is significant (c’ = −0.10; SE = 0.0456; *p* = 0.0271), which indicates that this is a partial mediation. In addition, the mother’s playful behavior had a significant positive effect on the child’s ER (b = 0.02; SE = 0.0061; *p* = 0.0000). Therefore, the results show that ES can directly predict child ER, and that child ER can also be predicted through mediated maternal playful behavior. The significance of indirect effects was determined through bias-corrected bootstrap confidence intervals using 5000 bootstrap samples and 95% confidence intervals. The indirect effect is significant (ab = −0.0543; Boot SE = 0.0262; CI [−0.1147; −0.0118]). Thus, the mother’s ER is significantly associated with the child’s ER. Specifically, the mother’s CR is positively associated with the child’s ER, while the mother’s ES is negatively associated with the child’s ER, as anticipated in H1. Moreover, maternal playful behavior mediates the association between maternal ER and child ER. Specifically, we anticipated that maternal CR will be positively associated with maternal playful behavior, which will further positively predict child ER, and that maternal ES will be negatively associated with maternal playful behavior, which will further predict child ER, as presumed in H2.

### 3.3. Moderated Mediation Analysis

**Figure 2:**
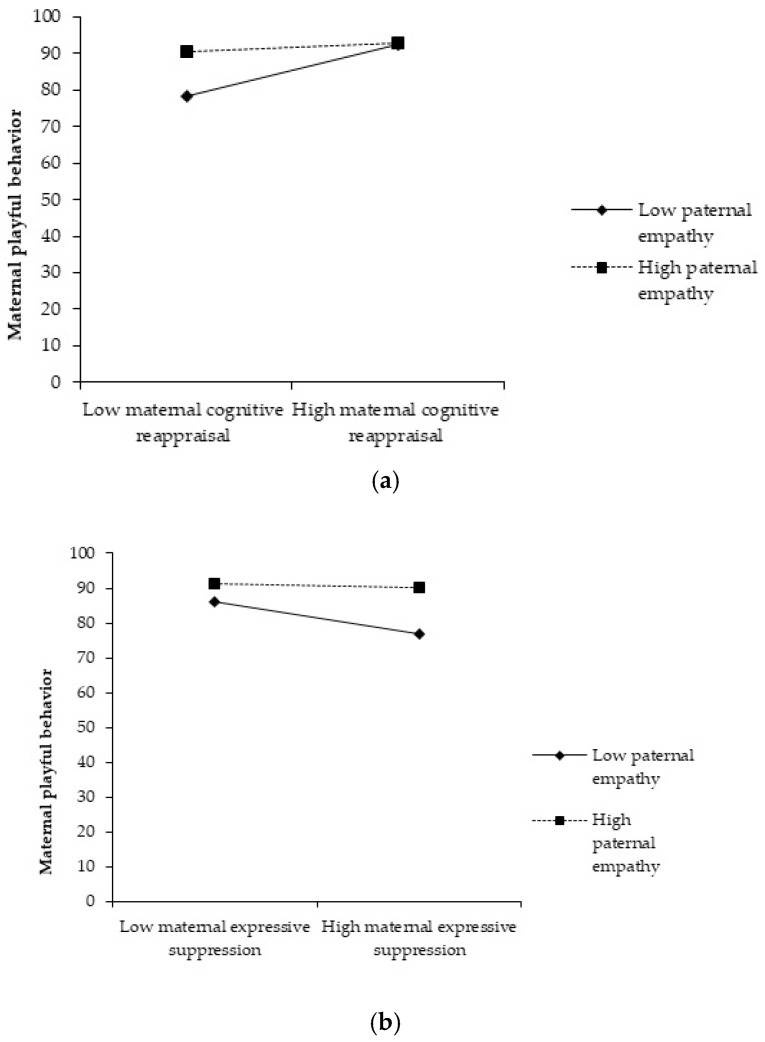
(**a**) A plot of the moderated relationship between maternal playful behavior and maternal cognitive reappraisal. (**b**) A plot of a moderated relationship between maternal playful behavior and maternal expressive suppression.

Furthermore, to test the conceptual moderated mediation model, we used the PROCESS v3.5 tool for IBM SPSS Model 7 [70]. To estimate 95% of the confidence intervals, we also used 5000 bootstrap samples by building bootstrap-based confidence intervals [71]. Specifically, we tested the interaction effect between maternal CR and paternal empathy on maternal playful behavior and the paths from maternal playful behavior to child ER and from maternal CR to child ER. We controlled the number of children, as well as the age and gender of the children. As expected, based on the results of previous mediation, CR did not predict child ER in the model where playful behavior was added (c’ = 0.0410; *p* = 0.5016) but positively predicted playful behavior (a = 15.3297; *p* = 0.0000). Furthermore, playful behavior positively predicted child ER (b = 0.0299; *p* = 0.0001). As such, the positive relation between maternal CR and child ER is fully mediated by maternal playful behavior. The complete results are shown in Table 5a. Also, the interaction effect of maternal CR and the partner’s empathy is significantly related to maternal playful behavior (int 0.1 = −0.1475; *p* = 0.004). The conditional indirect effect of the higher levels of paternal empathy (mean + 1SD) was insignificant, as the confidence interval included zero (CI [−0.0347; 0.1120]). The conditional indirect effect of the lower levels of paternal empathy (mean − 1SD) was significant, and the confidence interval of the indirect effect did not include zero (CI [0.0871; 0.2750]). As such, lower maternal CR levels were related to less playful behavior only when paternal empathy was low (Figure 2a). Therefore, the partner’s empathy moderates the relationship between maternal CR and playful behavior (H3). More details of mediation and moderation effects between maternal CR and child ER are presented in Table 6a.

Furthermore, we tested the interaction effect between maternal ES and paternal empathy on maternal playful behavior as well as the paths from maternal playful behavior to child ER and from maternal ES to child ER. Also, we controlled the number of children and the age and gender of the children. The results show that ES negatively predicted child ER in the model where maternal playful behavior was added (c’ = −0.1023; *p* = 0.0271) and negatively predicted playful behavior (a = −8.9821; *p* = 0.0029). Furthermore, playful behavior positively predicted child ER (b = 0.0293; *p* = 0.0000). As such, the negative relation between maternal ES and child ER is partially mediated by maternal playful behavior. The complete results are shown in Table 5b. Also, the interaction effect of maternal ES and the partner’s empathy is significantly related to maternal playful behavior (int 0.1 = 0.0899; *p* = 0.0120). The conditional indirect effect of higher levels of paternal empathy (mean + 1SD) was insignificant, as the confidence interval includes zero (CI [−0.0568; 0.0425]). The conditional indirect effect of the lower levels of paternal empathy was significant; the confidence interval of the indirect effect did not include zero (CI [−0.1735; −0.0341]). As such, higher levels of maternal ES were related to less playfulness only when paternal empathy was low (Figure 2b). More details of the mediation and moderation effects between maternal ES and child ER are presented in Table 6b.

The partner’s empathy moderates the relationship between maternal ES and the mother’s playful behavior (H3). More details of the mediation and moderation effects between maternal ES and child ER are presented in Table 6b.

## 4. Discussion

This study aimed to test the direct and indirect relationships in the association between mother and child ER. Specifically, we investigated the mediating role of maternal playful behavior and the moderating role of paternal empathy. The correlational analyses revealed that CR is an adaptive ER strategy associated with positive socio-emotional outcomes in children, whereas ES is a maladaptive strategy associated with poorer socio-emotional outcomes. Our correlational results reveal a positive association between maternal CR and a mother’s playful behavior and a negative association between maternal ES and a mother’s playful behavior. Also, a father’s empathy is positively associated with a mother’s CR, maternal playful behavior, and a mother’s perception of a child’s ER. As such, our findings align with other studies that show the positive implications of fathers’ empathy related to the children’s and mothers’ socio-emotional outcomes [72,73].

The association between mother ER and child ER

Previous studies have shown that parental CR is positively associated with child ER, whereas ES is negatively associated with child ER. Our findings support the first hypothesis and is in line with the results of previous studies, which revealed the negative consequences of ES on children’s socio-emotional outcomes [34,74] and the positive implications of mothers’ CR on children’s socio-emotional outcomes [75]. As such, CR is an adaptive strategy, inclusive in the parenting domain, not only in one of the adults’ social functioning domains. For example, a study that used both biological and behavioral indices found that parents’ CR, alongside a physical index (e.g., respiratory sinus arrhythmia), is associated with children’s adaptative physiological regulation, an indicator of children’s adaptative emotional regulation [76]. Regarding ES, although it is known that it is a maladaptive type of ER in general [77], it is also dysfunctional in the field of parenting, impairing children’s ER through the fact that parents who suppress their emotions are not able to model adaptative ER strategies to their children [78]. Moreover, the obtained results are explained through the components of the Tripartite Model of the Impact of the Family on Children’s Emotion Regulation [8], such as maternal modeling. As such, a mother’s ER is essential for a child’s ER, as preschoolers rely on extrinsic ER provided mainly by mothers. Therefore, the successful transition from extrinsic to intrinsic child ER depends on the mother’s ER strategies [77,79].

The mediating role of a mother’s playful behavior

The results confirm that maternal playful behavior mediates the relationship between mother CR, ES, and child ER, which confirms the second hypothesis. Mothers using CR are more willing to engage in playful interactions with their children, providing an important avenue when it comes to developing socio-emotional children. Mothers using CR engage in more playful interactions with their children since both reappraisal and playful behaviors involve reframing situations to reduce negative emotions and enhance positive ones [33,80]. Furthermore, previous research found that playfulness is associated with lower stress levels, efficient coping strategies, and less employment of negative and avoidant strategies among adults [81,82]. One of the aims of ER is to decrease the experience or expression of negative emotions [58]. Therefore, parental playful behavior creates a positive emotional climate favorable to a child’s ER development [8], resulting in a better ER [15,39]. Furthermore, in line with previous studies, ES is associated with less well-being, less optimism, and more negative emotions, with all of these negative outcomes being opposite to those that playfulness involves, such as enjoyment, fun, and, in general, a positive effect [33,37]. As such, parental ES reduces maternal playful interactions with children. Furthermore, reduced motherly playful behavior still promotes child ER, as playfulness is vital for children’s socio-emotional development [38]. These results are in accordance with the Tripartite Model [8] in which parental characteristics, along with parenting behavior (e.g., playful behavior), are an important factor in shaping children’s ER [62,63].

Moderating role of paternal empathy

Our results partially support our third hypothesis. The main finding of our study is that lower levels of paternal empathy moderate the association between a mother’s ER (CR and ES) and playful behavior. Until now, studies have investigated the ability of parents (especially mothers) to empathize with their children [73,83], and very few studies have investigated the role of paternal empathy on maternal variables’ variations, with a few exceptions [72]. Because empathy implies affective sharing, emotional availability, social interaction, and minimizing others’ felt distress, it has had a buffer effect on the mother’s ER difficulties elicited by the challenging task of childrearing. Therefore, paternal empathy creates the context for mothers’ better ER and well-being, as well as a high propensity to engage in playful behavior. The pressure of childrearing may impair mothers’ ER, leading to fewer emotional resources. Therefore, fathers with lower empathy may be perceived by mothers as emotionally unavailable, which is associated with lower levels of maternal playful behavior [84,85]. Our findings show that higher levels of empathy in partners does not moderate the association between a mother’s ER (CR and ES) and a mother’s playful behavior. In line with this result, Zhang and colleagues [47] did not find a moderating role of high levels versus low levels of emotional support (which includes empathy) between maternal parenting stress and playful mother–child interactions. On the other hand, besides the absence of the moderating effect, they found that mothers who received higher levels of emotional support from their partners, such as empathy [46], reported more playful mother–child interactions. Therefore, our results suggest that for mothers, lower levels of empathy from their partners are acutely felt rather than higher levels in the association between maternal ER and playful behavior. By studying the moderating effects of empathy, we provide theoretical support for the situations in which lower levels of paternal empathy will mitigate mothers’ playful behavior, namely when maternal CR is lower or when maternal ES is higher.

Limitations and Future Research Directions

First, the children’s ER was assessed by their mothers. Future studies should include additional assessments of children’s variables (e.g., teacher interviews and laboratory tasks). Second, we investigated the moderating role of paternal global empathy, including both cognitive and affective dimensions, based on their moderate correlation [69]. Future studies should explore both dimensions of empathy as moderators of the association between different maternal characteristics and maternal playful behavior. In addition, future studies can explore the moderating role of maternal empathy in the model testing the mediating role of paternal playful behavior in the association between paternal ER and the paternal perception of child ER. Third, all study variables were measured by self-report, and a cross-sectional study design was used. Future studies should include additional variable measurement methods, such as qualitative ones (e.g., interviews), or integrate observational measures of children’s self-regulation, such as the Head–Toes–Knees–Shoulders Task (HTKS) [86], and integrate a longitudinal design to establish a causal relation among the study’s variables. Finally, our sample size was relatively small due to the difficulty of gathering data from both partners. Our findings contribute to clinical therapy or psychological counseling regarding children’s emotional regulation development, where the focus should be on the entire family, including playful mother–child interactions and mother–father relationships [47].

## 5. Conclusions

Maternal CR is positively associated with child ER, whereas maternal ES is negatively associated with child ER. Maternal ER (both CR and ES) was directly and indirectly associated with child ER through maternal playful behavior, and the father’s empathy moderated the association between maternal ER and maternal playful behavior. Considering the important role of maternal playful behavior when it comes to promoting child ER, future studies should explore additional protective factors sustaining the mother’s playful behavior when interacting with her child. Our results also underline the indirect influence of fathers on children’s socio-emotional outcomes through their influence on mothers.

## Figures and Tables

**Table 1:** The socio-demographic characteristics of the participants (N = 103).

Variable	N	%	M	SD	Range
**Age**					
Mother’s age			34.93	4.78	25–52
Fathers’ age			37.52	4.57	28–54
Child’s age			4.73	1.21	3–6
**Child’s gender**					
Female	57	55.3%			
Male	46	44.7%			
**Family residence**					
Urban residence	66	64.1%			
Rural residence	37	35.9%			
**Parents’ education**					
**Post-academic degrees**					
Mothers	45	43.7%			
Fathers	27	26.2%			
**Bachelor’s degrees**					
Mothers	42	40.8%			
Fathers	40	38.8%			
**Secondary, post-secondary, or professional school**					
Mothers	16	15.5%			
Fathers	36	34.9%			

**Table 2:** Descriptive statistics for the main variables (N = 103).

Variable	M	SD	Min	Max	Skewness (SE)	Kurtosis (SE)
Cognitive reappraisal	4.82	1.22	2.33	7	−0.19 (0.23)	−0.81 (0.47)
Expressive suppression	3.41	1.40	1.25	7	0.41 (0.23)	−0.54 (0.47)
Partner’s empathy	80.19	15.80	44	110	−0.22 (0.23)	−0.58 (0.47)
Playful behavior	71.08	10.88	44	91	0.59 (0.23)	−0.27 (0.47)
Child ER	3.19	0.71	1.79	5	0.31 (0.23)	−0.15 (0.47)
Child’s age	4.71	1.21	3	6	−0.30 (0.23)	−1.49 (0.47)

**Table 3:** Associations between the main variables (N = 103).

Variables	1	2	3	4	5	6	7
1. Cognitive reappraisal	1						
2. Expressive suppression	0.03	1					
3. Partner’s empathy	0.40 **	−0.12	1				
4. Playful behavior	0.53 **	−0.37 **	0.54 **	1			
5. Child ER	0.38 **	−0.33 **	0.37 **	0.48 **	1		
6. Child’s age	0.02	0.29 **	−0.07	−0.33 **	0.04	1	
7. Number of children	0.26 **	−0.11	0.17	0.14	0.32 **	0.09	1
8. Child’s gender	−0.10	0.14	−0.13	−0.19 *	−0.20 *	−0.15	−0.09

Note. * *p* < 0.05; ** *p* < 0.01.

**Table 4:** Direct, indirect, and total effects.

Cognitive reappraisal (a path)	Maternal playful behavior as outcome			
	Coefficient	SE	*t*	*p*
	4.65	0.6906	6.74	0.0000
Expressive suppression (a path)	−1.85	0.729	−2.54	0.0124
	Child emotion regulation as outcome			
	Coefficient	SE	*t*	*p*
Maternal playful behavior (b path)	0.02	0.0073	4.07	0.0001
Cognitive reappraisal (c’ direct path)	0.04	0.0607	0.6745	0.5016
Cognitive reappraisal (c total path)	0.1803	0.0540	3.3364	0.0012
Confidence Interval 95%				
Cognitive reappraisal * playful behavior (indirect a * b path)		Lower Limit		Upper limit
	0.1393	0.0676		0.2353
	Child emotion regulation as outcome			
	Coefficient	SE	*t*	*p*
Maternal playful behavior (b path)	0.02	0.0061	4.7819	0.000
Expressive suppression (c’ direct path)	−0.10	0.0456	−2.2434	0.0271
Expressive suppression (c total path)	−0.15	0.0489	−3.2067	0.0018
Confidence Interval 95%				
Expressive suppression * playful behavior (indirect a * b path)		Lower Limit		Upper limit
	−0.0543	−0.1147		−0.0188

**Table 5:** (**a**) Path estimates for testing the moderation and mediation model between mother CR and child ER. (**b**) Path estimates for testing the moderation and mediation model between mother ES and child ER.

(**a**)				
	**Coefficient**	**SE**	***t***	***p***
Playful behavior as outcome (M)				
Cognitive reappraisal	15.3297	3.2955	4.65	0.0000
Partner’s empathy	0.9244	0.1957	4.72	0.0000
**Interaction 1**	−0.1475	0.0404	−3.6528	0.0004
Child ER as an outcome (DV)				
Cognitive reappraisal	0.0410	0.0607	0.67	0.5016
Playful behavior	0.0299	0.0073	4.07	0.0001
Number of children as a covariate	0.2437	0.0991	2.4590	0.0157
(**b**)				
	**Coefficient**	**SE**	***t***	***p***
Playful behavior as outcome (Mediator)				
Expressive suppresion	−8.9821	2.9364	−3.0588	0.0029
Partner’s empathy	−0.0147	0.1419	−0.1036	0.9177
**Interaction 1**	0.0899	0.0351	2.5614	0.0120
Child ER as outcome (DV)				
Expressive suppresion	−0.1023	0.0456	−2.2434	0.0271
Playful behavior	0.0293	0.0061	4.7819	0.0000
Number of children	0.2374	0.0953	2.4904	0.0145
Child’s age	0.1336	0.536	2.4898	0.0145

**Table 6:** (**a**) Mediation and moderation effects between mother CR and child ER. (**b**) Mediation and moderation effects between mother ES and child ER.

(**a**)				
	**Coefficient**	**SE**	**Confidence Interval 95%**	
			Lower Limit	Upper Limit
Mediation				
Playful behavior	0.1393 ^a^	0.0429	0.0676	0.2353
Moderated mediation				
Partner’s empathy	−0.0044 ^b^	0.0017	−0.0079	−0.0015
Number of children	0.2437	0.0991	0.0470	0.4403
(**b**)				
	**Coefficient**	**SE**	**Confidence Interval 95%**	
			Lower Limit	Upper Limit
Mediation				
Playful behavior	−0.0543 ^c^	0.0262	−0.1147	−0.0118
Moderated mediation				
Partner’s empathy	0.0026 ^b^	0.0012	0.0006	0.0053
Number of children	0.2374	0.0953	0.0482	0.4266
Child’s age	0.1336	0.0536	0.0271	0.2400

Note. ^a^ Indirect effect of CR on child ER; ^b^ index of moderated mediation; ^c^ indirect effect of ES on child ER.

## Data Availability

The data that support the findings of this study are available upon request from the corresponding author due to privacy restrictions.
